# COVID-19 and silent hypoxemia in a minimal closed-loop model of the respiratory rhythm generator

**DOI:** 10.1007/s00422-024-00989-w

**Published:** 2024-06-17

**Authors:** Casey O. Diekman, Peter J. Thomas, Christopher G. Wilson

**Affiliations:** 1 Department of Mathematical Sciences, New Jersey Institute of Technology, University Heights, Newark, NJ 07102 USA; 2 Department of Mathematics, Applied Mathematics and Statistics, Case Western Reserve University, 10900 Euclid Avenue, Cleveland, OH 44106 USA; 3 Department of Pediatrics and Basic Sciences, Lawrence D. Longo, MD Center for Perinatal Biology, Loma Linda University, 11223 Campus St, Loma Linda, CA 92350 USA

**Keywords:** Silent hypoxemia, Breathing control, Central pattern generator, Computational modeling, COVID-19, Polycythemia, Sensory feedback

## Abstract

Silent hypoxemia, or “happy hypoxia,” is a puzzling phenomenon in which patients who have contracted COVID-19 exhibit very low oxygen saturation ($\text {SaO}_2$ < 80%) but do not experience discomfort in breathing. The mechanism by which this blunted response to hypoxia occurs is unknown. We have previously shown that a computational model of the respiratory neural network (Diekman et al. in J Neurophysiol 118(4):2194–2215, 2017) can be used to test hypotheses focused on changes in chemosensory inputs to the central pattern generator (CPG). We hypothesize that altered chemosensory function at the level of the carotid bodies and/or the *nucleus tractus solitarii* are responsible for the blunted response to hypoxia. Here, we use our model to explore this hypothesis by altering the properties of the gain function representing oxygen sensing inputs to the CPG. We then vary other parameters in the model and show that oxygen carrying capacity is the most salient factor for producing silent hypoxemia. We call for clinicians to measure hematocrit as a clinical index of altered physiology in response to COVID-19 infection.

## Introduction

### Background

The global COVID-19 pandemic led to over 1,003,000 deaths in the USA, and over 6,881,000 worldwide, from its onset in late 2019 through March, 2023 (Johns Hopkins University Coronavirus Research Center 2023). COVID-19 can cause profoundly low levels of oxygen in the blood (hypoxemia) with near normal arterial carbon dioxide ($P_{\textrm{a}}\text {CO}_2$) levels. Although some individuals with COVID-19-induced hypoxemia experience dyspnea (breathing discomfort), many do not (Dhont et al. 2020). During surges of the pandemic, patients arriving at already overcrowded emergency rooms (ERs) who were not in obvious respiratory distress were often triaged (Dhont et al. 2020). However, some of these patients may have had reduced oxygen saturation despite their lack of dyspnea (Simonson et al. 2021; Berezin et al. 2021; Chandra et al. 2020). This subpopulation of COVID-19 patients present with a novel condition known as *silent hypoxemia* or “happy hypoxia” (Simonson et al. 2021).

Silent hypoxemia can result in tachypnea (rapid, shallow breathing), and with severe hypoxemia, changes in ventilation can occur (Easton et al. 1986; Easton and Anthonisen 1988), but in general there is an absence of increased alveolar ventilation (Dhont et al. 2020). The mechanism underlying this condition is poorly understood but has been hypothesized to depend upon high expression levels of angiotensin converting enzyme 2 (ACE2) in the lungs, carotid body, and, perhaps, in the central breathing control circuitry within the medulla oblongata (Simonson et al. 2021). ACE2 is the cellular entry point for SARS-CoV-2 (Yuki et al. 2020). Additionally, recent work has shown that there is a shift in the oxyhemoglobin dissociation curve^1^ in COVID-19 patients (Vogel et al. 2020; Ceruti et al. 2022). Since carotid body chemoreceptors respond to both low O_2_ and high CO_2_, a primary problem in these patients may be dysregulation of these sensors and chemosensory reflexes in general. COVID-19 infection has been shown to increase ACE2 expression, leading to changes in sensitivity to both CO_2_ and O_2_; changes in blood gases lead to a concomitant change in activity within the *nucleus tractus solitarii* (NTS). Recent work has shown that ACE2 is present within the carotid bodies of humans (Porzionato et al. 2021; Villadiego et al. 2021) and there is evidence of altered chemosensation across multiple systems with SARS-CoV-2 infection (Caretta and Mucignat-Caretta 2022). The absence of dyspnea—even though patients exhibit low oxygen saturation—suggests that changes in carotid body inputs to the NTS are a key feature of SARS-CoV-2 infection. Additionally, there may be changes in NTS activity that contribute to the blunted ventilatory response but this has not yet been reported.

### Altered chemosensory function and silent hypoxemia

After four years of the COVID-19 pandemic and ongoing endemic infection, a few key pathophysiologies have become apparent. First, ACE2 expression is correlated with the location and severity of infection (Zou et al. 2020). Because ACE2 is, based on current knowledge, the main vector by which SARS-CoV-2 enters the body’s cells, changes in ACE2 expression should have an impact on the severity and time course of COVID-19 symptoms. Second, changes in NTS signaling may play a key role in altering the normal, physiological response to changes in oxygenation during COVID-19, and that information may be carried by the glossopharyngeal nerve (innervating the carotid body) or lung afferents via the vagus nerve. Information sensed at the carotid bodies (and lung interoceptors) ultimately reaches the NTS via the vagus and glossopharyngeal nerves. From the NTS, these signals are distributed to local visceral integration circuits within the medulla, including the cardivascular control regions (rostral and caudal in the ventral medulla) and the preBötzinger complex and associated regions of respiratory control within the brainstem.

Based on the clinical observations reported so far, it appears that there is a change in gain in the pathway from carotid body, to NTS, to the breathing rhythm generator and pattern formation network. These observations in patients have provided the motivation for us to focus on assessing the effect of changes in sensitivity/gain in this signaling pathway. This change in gain may be more prevalent in any one of these circuit elements and further work needs to be done to determine the exact mechanism by which sensitivity of the control circuit is impacted.

Given the low partial pressure of oxygen in arterial blood ($P_{\textrm{a}}\text {O}_2$) of patients infected with SARS-CoV-2 virus (Sartini et al. 2020; Chen et al. 2020) and the high expression of ACE2 in the carotid bodies, it is likely that altered chemosensory reflexes play a central role in the symptoms and outcomes seen in COVID-19 patients (Porzionato et al. 2021, 2020). In light of this data, we hypothesize that altered chemosensory function at the level of the carotid bodies and/or the NTS are responsible for this blunted response to hypoxia.

### Overview of our approach

We use a previously published computational model of respiratory control (Diekman et al. 2017) to explore this hypothesis by altering the properties of the gain function representing oxygen sensing inputs to the respiratory central pattern generator (CPG). As reviewed in Sect. 4, there are several respiratory control models featuring sensitivity to hypercapnia, which under normal circumstances plays the leading role in regulating breathing effort, and few models based on hypoxia-driven chemosensory feedback. Because hypoxia seems to coexist with normal CO_2_ levels in silent hypoxemia, we base our investigation on a closed-loop respiratory control model focused on blood oxygen regulation. The respiratory control model studied in Diekman et al. (2017) has seven dynamical variables: voltage of a central pacemaker cell, together with one fast and one slow gating variable; diaphragm muscle activation; lung volume; partial pressure of O_2_ in the lung; and partial pressure of O_2_ in the bloodstream. Regulation of the endogenous breathing rhythm occurs through hypoxia-sensitive chemosensory feedback in the model. Thus, we refer to this system as the 7D-O2 model. The 7D-O2 model strikes a balance between simplicity, in order to preserve analytic transparency, and complexity, in order to capture the phenomenon of interest. See Sect. 4 for further discussion about the realism/tractability trade-off in modeling respiratory control.

**Fig. 1:**
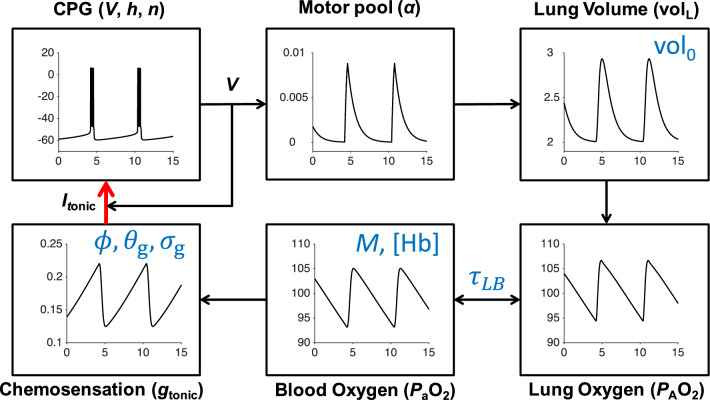
Schematic of the 7D-O2 model. Bursts of action potential firing (*V*, mV) in the respiratory central pattern generator (CPG) drive a pool of motor neurons ($\alpha$, dimensionless), leading to expansions of lung volume (vol_L_, L) and increases in lung and blood oxygen ($P_{\textrm{A}}\textrm{O}_2$ and $P_{\textrm{a}}\textrm{O}_2$, mmHg). Through a chemosensory pathway ($g_{\textrm{tonic}}$, nS), the blood oxygen level affects the amount of excitatory current sent to the CPG, thereby closing the control loop (red arrow). Time (*t*, seconds) is the horizontal axis for all traces. The seven parameters shown in blue (chemosensory feedback parameters $\phi$, $\theta _{\textrm{g}}$, and $\sigma _{\textrm{g}}$, see Methods; hemoglobin concentration [Hb]; base lung volume $\text {vol}_0$; time constant $\tau _{\textrm{LB}}$; and metabolic demand parameter *M*) are varied in this study to model silent hypoxemia. Redrawn, with modifications, from Diekman et al. (2017)

In this paper, we use the 7D-O2 model to explore our hypothesis by altering the properties of the gain function representing oxygen sensing inputs to the CPG. We then vary other parameters in the model, and show that oxygen carrying capacity is the most salient factor for producing silent hypoxemia. We exploit the presence of a small parameter (the Henry’s Law constant) in the expression for the O_2_ saturation curve to provide a mathematical explanation for the effect of changing the hemoglobin concentration (hematocrit) in the model.^2^.

**Fig. 2:**
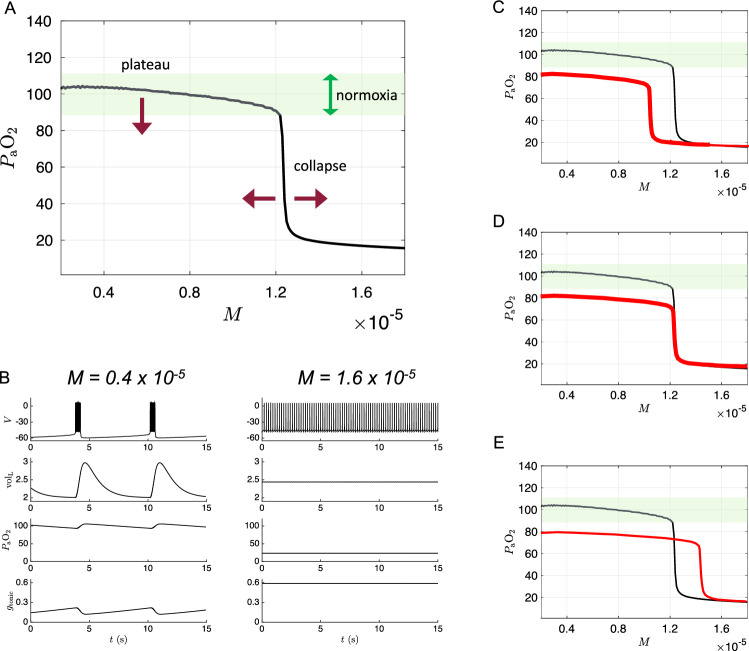
Dynamics of 7D-O2 model and hypothetical silent hypoxemia $P_{\textrm{a}}\text {O}_2$ versus *M* curves. **a** Average blood oxygen $P_{\textrm{a}}\text {O}_2$ as a function of metabolic demand (*M*) in the original 7D-O2 model. There is a plateau region at low *M* values for which normoxia (green shading) is maintained, and a collapse point at approximately $M=1.2\times 10^{-5}$ ms^-1^ beyond which severe hypoxia occurs. In a model of silent hypoxemia, it seems clear that the plateau portion of the curve should shift lower (maroon arrow pointing down), but it is not as clear whether the collapse point should remain in the same location or shift horizontally (maroon arrows pointing left and right). **b** Variables of the 7D-O2 model for *M* values in the plateau region (left column) or after the collapse point (right column). **c–e** Hypothetical silent hypoxemia models (red) with a lower plateau and a collapse point shifted to a lower *M* value (**c**), in the same location (**d**), or shifted to a higher *M* value (**e**) compared to the 7D-O2 model (black)

Figure 1 shows a schematic of the 7D-O2 model with components representing CPG membrane potential (*V*), motor pool activity ($\alpha$), lung volume ($\text {vol}_{\textrm{L}}$), lung oxygen ($P_{\textrm{A}}\text {O}_2$), blood oxygen ($P_{\textrm{a}}\text {O}_2$), and chemosensation ($g_{\textrm{tonic}}$). The model has a closed-loop structure since an excitatory current, $I_{\textrm{tonic}}$, depends on $P_{\textrm{a}}\text {O}_2$ and is an input to the CPG component (red arrow). In this model, the rate of metabolic demand for oxygen from the tissues is represented by the parameter *M*. If metabolic demand is low or moderate ($M<1.2\times 10^{-5}$ ms^-1^), then the model exhibits a stable eupneic rhythm with CPG bursting activity driving fluctuations in lung volume that bring in a sufficient amount of oxygen to maintain $P_{\textrm{a}}\text {O}_2$ in the normoxia range (see the “plateau” region of the $P_{\textrm{a}}\text {O}_2$ versus *M* curve shown in Fig. 2a and the traces in the left panel of Fig. 2b.) However, if metabolic demand is too high ($M>1.2\times 10^{-5})$, then the model exhibits a form of tachypnea that transitions to tonic CPG bursting activity that does not drive the lungs to effectively maintain $P_{\textrm{a}}\text {O}_2$ in the normoxia range (see the “collapse” region of Fig. 2a and the right panel of Fig. 2b).

In silent hypoxemia, we would expect to observe a lower height for the plateau region of the $P_{\textrm{a}}\text {O}_2$ versus *M* curve, since these patients display abnormally low $P_{\textrm{a}}\text {O}_2$ despite minimal changes in minute ventilation. There are three possibilities regarding the collapse region in silent hypoxemia patients: the collapse point could shift to a lower *M* value (as illustrated in Fig. 2c), stay at the same *M* value (as in Fig. 2d), or shift to a higher *M* value (as in Fig. 2e). Due to an acute disease-induced reduction in steady-state $P_{\textrm{a}}\text {O}_2$, it seems plausible that there would be a *decrease* in tolerance of higher metabolic demand. Therefore, we explored parameter space to see if the closed-loop model is capable of producing $P_{\textrm{a}}\text {O}_2$ versus *M* curves with shapes similar to the hypothetical curve shown in Fig. 2c.

## Methods

In this section, we will briefly describe the 7D-O2 model. For a full description of the nonlinear system of seven ordinary differential equations specifying the model, see the “Appendix A.1”.

### Quantitative modeling approach

Quantitative modeling has helped elucidate principles of normal and pathological functioning of the respiratory system, although its fundamental mechanisms remain debated. Mathematical models can be particularly helpful for generating experimentally testable hypotheses. A variety of models have been developed for the respiratory CPG (Butera et al. 1999a, b; Del Negro et al. 2002; Rubin 2008; Del Negro and Hayes 2008; Rubin et al. 2009; Phillips and Rubin 2019; Phillips et al. 2022), for chemosensory feedback-based regulation schemes (Grodins et al. 1954; Khoo et al. 1982; Cherniack and Longobardo 2006), and for cardiopulmonary gas exchange (Ben-Tal 2006). See Molkov et al. (2017) and Lindsey et al. (2012) for a review. A smaller number of published models represent closed-loop control incorporating a conductance-based CPG, muscle dynamics, gas exchange, and sensory feedback (Ben-Tal and Smith 2008; Park et al. 2012; Molkov et al. 2014). Of these, several focus on hypercapnia (excessive CO_2_) as the regulatory pathway. In order to generate hypotheses about silent hypoxemia, we chose to work with a conductance-based CPG model with O_2_ chemosensation as the sensory feedback pathway closing the control loop. To our knowledge, our previously published model (Diekman et al. 2017) is the only model meeting these criteria. Aspects of it have been experimentally validated (Diekman et al 2022; Diekman et al. 2018). Like any model, this model fails to represent all aspects of the control system. We have not included CO_2_ sensing in our model due to the high diffusion rates of CO_2_ when compared to O_2_ in the lung (West 2008) and evidence showing that CO_2_ is $\le$ 35 mmHg in patients presenting with silent hypoxemia (SH) and minimal tachypnea (Chandra et al. 2020; Alamé et al. 2022). Additionally, we do not explicitly include rapidly adapting (RAR) or slowly adapting (SAR) lung mechanoreceptors in the model—lung volume is present in the model and reproduces inspiratory drive in much the same way that SARs do in vivo. Nevertheless, in spite of these limitations, the model suffices to generate testable hypotheses that could be pursued by the clinical community.

The 7D-O2 model is a closed-loop respiratory control model that comprises a well-established conductance-based central rhythm generator (the Butera–Rinzel–Smith model (Butera et al. 1999a; Diekman et al. 2017)) with a voltage variable *V*, a fast gating variable (delayed-rectifier potassium current activation, *n*), and a slow gating variable (persistent sodium current inactivation, *h*). The output of the BRS model cell, namely the voltage, drives a motor pool activation variable, $\alpha$, that in turn drives expansion of the lungs. The lung volume ($\text {vol}_{\textrm{L}}$), the partial pressure of oxygen in the lungs (alveolar pressure, $P_{\textrm{A}}\text {O}_2$), and the partial pressure of oxygen in the bloodstream ($P_{\textrm{a}}\text {O}_2$) complete the model variables. The BRS cell includes an excitatory current driven by a tonic conductance that is regulated by chemosensory feedback, closing the control loop. When the tonic conductance assumes intermediate values, the BRS cell exhibits bursting activity, consistent with eupnea (normal steady breathing). If blood O_2_ levels are significantly reduced, the tonic conductance increases, which can trigger a transition into a rapid, tonically firing “beating” regime, analogous to tachypnea (pathologically rapid shallow breathing). If blood O_2_ levels are significantly increased, the tonic conductance decreases, which can push the BRS model cell into a stable resting fixed point at, which Butera et al. called the “quiescent” regime (Butera et al. 1999a). The 7D-O2 model includes a metabolic demand parameter, *M*, regulating the rate at which oxygen is removed from the bloodstream to the tissues. As the “phenotype” or “physiology” of the model, we take the steady-state value of $P_{\textrm{a}}\text {O}_2$ as a function of *M*. For the original model as presented in Diekman et al. (2017), the $P_{\textrm{a}}\text {O}_2$-vs-*M* curve shows a plateau near 100 mm Hg (normoxia) that collapses to a critically hypoxic state when *M* increases past a high threshold (Fig. 2a). As we varied the original parameters to investigate possible mechanisms of silent hypoxemia, we monitored the height of the normoxia plateau, and the location of the collapse point.

Simulations were conducted using MATLAB v. 2020B on the NJIT high-performance computing cluster. Code corresponding to the 7D-O2 model is posted on Github at https://github.com/ModelDBRepository/229640 and on ModelDB at https://modeldb.science/229640. Code corresponding to the silent hypoxemia model developed here is available at https://modeldb.science/2015954.

### Relating model parameters to potential silent hypoxemia mechanisms

The mechanism by which COVID-19 leads to sustained hypoxemia in the absence of dyspnea is currently unknown. The minimalist model of Diekman et al. (2017) includes a number of key parameters that are plausible targets for modification to mimic the effects of COVID-19-infection on respiratory control.

Oxygen carrying capacity is a key variable in pulmonary mechanics. Repeated bouts of intermittent hypoxia, as seen in obstructive sleep apnea, can increase HIF-1$\alpha$ signaling, with a subsequent increase in erythropoietin, and an increase in hemoglobin and erythrocytes. Similar changes are seen in conditions that result in chronic hypoxemia and hypercapnia, such as cardiovascular disease, obstructive sleep apnea, and chronic obstructive pulmonary disease (Mauad et al. 2021; Paquette et al. 2021; Li et al. 2019). Many of the patients presenting with silent hypoxemia have pre-existing conditions and comorbidities that are likely to increase hematocrit and this increase in oxygen carrying capacity may blunt chemoreceptor responses—exacerbating the “happy hypoxia” phenomenon. Unfortunately, no current literature quantifies hematocrit in these patients.

Motivated by these observations, we systematically varied (plus or minus 20%) the following parameters that control the saturating effect of hypoxia-sensitive chemosensory feedback to the central pattern generator: $\sigma _{\textrm{g}}$, which controls the slope of the sensory feedback curve at maximum sensitivity (gain at threshold); $\theta _{\textrm{g}}$, which controls the threshold activation value for sensory feedback (50% activation point); and $\phi$, which controls the maximum sensory feedback drive at full activation.

Lung volume is a key determinant of mechanosensory feedback to the NTS and the CPG. Our model incorporates lung volume and allows us to monitor changes in lung volume in response to changes in central drive for breathing. This also allows us to monitor lung volume as an outcome measure to determine if the CPG is actually causing lung inflation in a way that assures sufficient gas exchange to sustain life when extrapolated to animal models or human subjects.

Ventilation/perfusion matching is a key drive for respiration. In mammals, the interplay between cardiovascular and respiratory control is essential for ensuring that sufficient oxygen is delivered to the body and CO_2_ is removed via the lung. We have included a time constant for O_2_ transport between the lung and blood which allows us to simulate changes in diffusion and dwell time within the lung that correlate with diseases such as chronic obstructive pulmonary disease (COPD) and lung fibrosis. Oxygen consumption and CO_2_ production are key elements for determining how changes in breathing can match metabolic demand. We have included a simplified treatment of metabolism in the model. As a “biomarker” to test the model behavior, for all parameter sets we varied the metabolic demand parameter *M* across a range of values. We have not included CO_2_ in this model, because CO_2_ diffuses up to 20 times faster than O_2_ (West 2008) and patients with SH do not appear to be hypercapnic since there is very little change in breathing rate—CO_2_ is a potent stimulator of minute ventilation and hypercapnia results in pronounced increases in breathing frequency (Moosavi et al. 2003; Parshall et al. 2012; Nakano et al. 2015).

Additionally, we vary the hemoglobin concentration to mimic the effect of chronic hypoxia seen in humans living in hypoxic environments which can include mountain dwellers (Hancco et al. 2020), individuals with severe obstructive sleep apnea (Li et al. 2019), or other cardio-respiratory disorders (Paquette et al. 2021; Balasubramanian et al. 2021). These individuals can have high hematocrit, a corresponding increase in red blood cells, and increased blood viscosity—similar to what has been reported in COVID-19 patients (Choi et al. 2022).

## Results

### Twenty percent variation in parameters specifying chemosensory feedback gain is insufficient to qualitatively reproduce silent hypoxemia

**Fig. 3:**
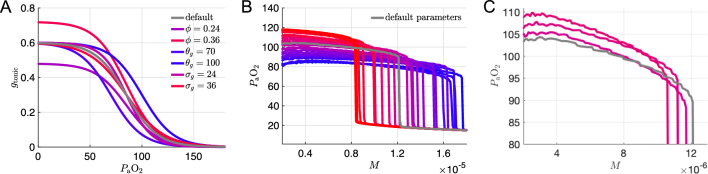
Sensitivity of $P_{\textrm{a}}\text {O}_2$ versus *M* curves to variation of model parameters. **a** Chemosensory sigmoid of $g_{\textrm{tonic}}$ as a function of $P_{\textrm{a}}\text {O}_2$ with various parameter values for the maximum ($\phi$), half-activation ($\theta _g$), and slope ($\sigma _g$) of the sigmoid. Default settings from the original 7D-O2 model ($\phi =0.3$ nS, $\theta _g=85$ mmHg, $\sigma _g=30$ mmHg) are shown in gray. See panel (**b**) for the definition of the color scale used for the other curves. **b** Average $P_{\textrm{a}}\text {O}_2$ vs *M* curves for 25 different combinations of the chemosensory sigmoid parameters ($\phi =0.24,0.3,0.36$; $\theta _g=70,85,100$; $\sigma _g=24,30,36$) on a color scale with the lowest and highest maximum $P_{\textrm{a}}\text {O}_2$ values are shown in blue and red, respectively, with the exception of the default parameter set which is shown in gray. Two of the 27 combinations were omitted for clarity. **c** Expanded view of curves from **b** close to the default curve. Parameter values $(\phi ,\theta _g,\sigma _g)$, listed from top to bottom at $M=4\times 10^{-6}$: (0.36, 85, 36), (0.36, 85, 30), (0.3, 85, 36); gray curve has the default values (0.3, 85, 30)

Motivated by the hypothesis that silent hypoxemia could result from a dysregulation of carotid body $\text {O}_2$ receptors, we first considered variation of the parameters associated with the chemosensory pathway of the model. In the 7D-O2 model, there is a sigmoidal relationship between $P_{\textrm{a}}\text {O}_2$ and $g_{\textrm{tonic}}$, with the parameters $\phi$, $\theta _g$, and $\sigma _g$ controlling the height, half-activation, and slope of the sigmoid, respectively (see Fig. 3a). We simulated the closed-loop model over a range of *M* values while varying these parameters over three levels spanning roughly $\pm 20\%$ of their original values ($\phi =0.24,0.3,0.36$, $\theta _g=70,85,100$, and $\sigma _g=0.24,0.3,0.36$), yielding 27 different combinations in total. Figure 3b shows that varying these parameters generates $P_{\textrm{a}}\text {O}_2$ vs *M* curves in which the plateau and collapse point are shifted down and to the right (similar to the hypothetical case shown in Fig. 2e). None of the 27 combinations, however, produce any curves with the plateau and the collapse point shifted down and to the left (similar to Fig. 2c). In patients with comorbidities that cause compensatory adaptations to chronic hypoxia—downstream from hypoxia-inducible factor 1$\alpha$ (HIF-1$\alpha$)—it seems plausible that disease-induced reduction in steady-state $P_{\textrm{a}}\text {O}_2$ would be accompanied by an *increase* in tolerance of higher metabolic demand. However, we do not consider any of these model variants to suitably capture the phenomenon of silent hypoxemia.

**Fig. 4:**
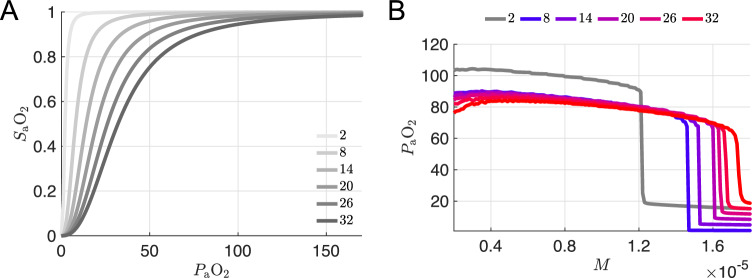
**a** Hemoglobin saturation curves (Eq. 18) for various hemoglobin binding affinities *K*. Default model has $K=26$ mmHg. **b** Average $P_{\textrm{a}}\text {O}_2$ vs *M* curves for the set of *K* values shown in (**a**)

In order to proceed further, we selected a single parameter set from among the 27 combinations previously considered as our working model for producing the hypoxic plateau region, namely $\phi =0.24$, $\theta _g=70$, and $\sigma _g=36$. These parameters gave the curve with the greatest reduction of $P_{\textrm{a}}\text {O}_2$ (darkest blue curves in Fig. 3a, b), although the collapse point did shift to significantly higher values of *M*. Next, based on reports indicating that COVID-19 patients have altered oxyhemoglobin dissociation curves (Vogel et al. 2020; Ceruti et al. 2022), we considered variation of the model parameter *K* which represents hemoglobin binding affinity (Eq. 18 in Methods).

### Varying the shape of the hemoglobin saturation curve leaves blood oxygen unchanged and weakly shifts the metabolic collapse point

The effect that increasing the binding affinity (decreasing *K*) has on the hemoglobin saturation versus blood oxygen curve ($\text {SaO}_2-P_{\textrm{a}}\text {O}_2$) with the new chemosensory parameters is shown in Fig. 4a. Tighter binding affinities (*K* values less than the default value of 26 mmHg) do shift the $P_{\textrm{a}}\text {O}_2$ vs *M* curve to the left, but the respiratory collapse point is still at higher metabolic demand values than the original model (Fig. 4b). See also Fig. 9a in “Appendix A.2” for the effect of varying *K* with the original chemosensory parameters.

Since varying the chemosensory parameters alone was not sufficient to model a silent hypoxemia patient prone to respiratory collapse, we considered other parameters that could plausibly be affected by COVID-19. Specifically, lung damage due to excessive immune response or local thrombosis could reduce the effective unloaded lung volume (model parameter $\text {vol}_0$), or impede the flux of oxygen between the alveoli and the alveolar capillaries. The latter effect could be reflected by an increase in the model parameter $\tau _{\textrm{LB}}$, which governs the effective relaxation time for differences in partial pressure of oxygen in the model’s lung and blood compartments, respectively. Therefore, while keeping the chemosensory sigmoid parameters $(\phi =0.24,\,\theta _g=70,\,\sigma _g=36)$ fixed, we varied the unloaded lung volume ($\text {vol}_0=1.6, 2.0, 2.4$) and the time constant for the flux of oxygen from the lung to the blood ($\tau _{\textrm{LB}}=100,500,900$).

**Fig. 5:**
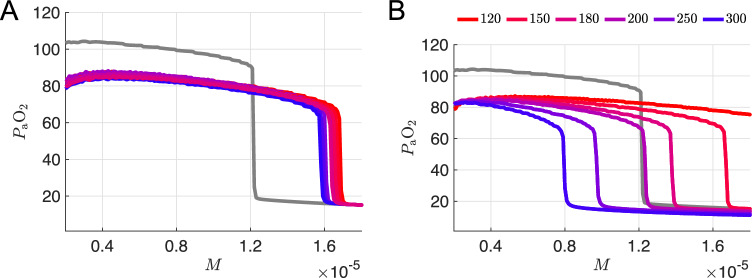
**a** Average $P_{\textrm{a}}\text {O}_2$ vs *M* curves for nine different combinations of oxygen flux and lung volume parameters ($\tau _{\textrm{LB}}=100,500,900$ ms; $\textrm{vol}_0=1.6,2.0,2.4$ L), with a constant set of chemosensory sigmoid parameters ($\phi =0.24$, $\theta _g=70$, $\sigma _g=36$) on a color scale with the lowest and highest *M* values at the collapse point ($P_{\textrm{a}}\text {O}_2=40$), are shown in blue and red, respectively, with the exception of the default parameter set which is shown in gray. **b** Average $P_{\textrm{a}}\text {O}_2$ vs *M* curves for six different values of hemoglobin concentration [Hb] (specifically [Hb]=120, 150, 180, 200, 250, 300 mmHg), with $\tau _{\textrm{LB}}=500$, $\textrm{vol}_0=2.0$, and the same chemosensory sigmoid parameters and color scale as in panel **a**. The purple $\mathrm{[Hb]}=250$ curve was selected as a putative model for silent hypoxemia

**Fig. 6:**
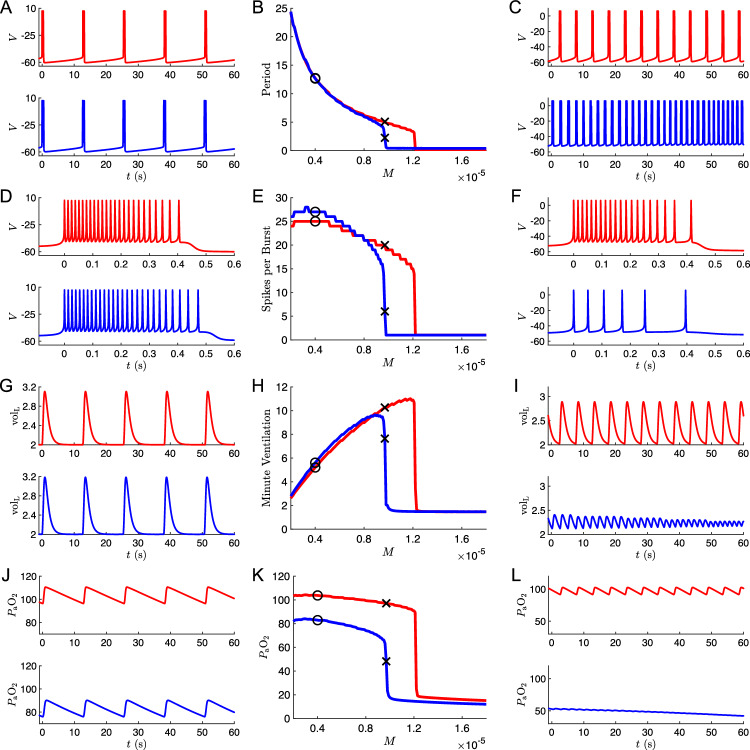
Simulations of putative silent hypoxemia model. **a–l** Output from simulations of the normoxia model (the original 7D-O2 model, red traces) and the silent hypoxemia model ($\mathrm{[Hb]}=250$ curve from Fig. 5b, blue traces). **a** Voltage traces showing multiple bursts for $M=0.4\times 10^{-5}\text {ms}^{-1}$. **b** Burst-to-burst period *T* as a function of metabolic demand *M*. Black markers indicate values of *M* used for panels (a,d,g,j) ($M=0.4\times 10^{-5}\text {ms}^{-1}$, black circles) and (c,f,i,l) ($M=0.97\times 10^{-5}\text {ms}^{-1}$, black Xs). **c** Voltage traces showing multiple bursts for $M=0.97\times 10^{-5}\text {ms}^{-1}$. **d** Voltage traces from (**a**) zoomed in on a single burst. **e** Spikes per burst as a function of *M*. **f** Voltage traces from (**c**) zoomed in on a single burst. **g** Lung volume for $M=0.4\times 10^{-5}\text {ms}^{-1}$. **h** Minute ventilation as a function of *M*. See text for details. **i** Lung volume for $M=0.97\times 10^{-5}\text {ms}^{-1}$. **j** Blood O2 traces across multiple bursts for $M=0.4\times 10^{-5}\text {ms}^{-1}$. **k** Average $P_{\textrm{a}}\text {O}_2$ as a function of *M*. **l** Blood O2 traces across multiple bursts for $M=0.97\times 10^{-5}\text {ms}^{-1}$

### Varying both oxygen flux and lung volume has little effect on the blood oxygen vs metabolic demand curve

As shown in Fig. 5a, varying $\text {vol}_0$ by $\pm 20\%$ and varying $\tau _{\textrm{LB}}$ by $\pm 400\%$ had surprisingly little effect on the height of the $P_{\textrm{a}}\text {O}_2$ versus *M* plateau, and did not significantly affect the collapse point either.

Having experimented with varying parameters specifying the shape of the chemosensory feedback response to hypoxia, the hemoglobin binding affinity constant *K*, and oxygen flux and lung volume parameters, we were able to significantly reduce the height of the eupneic $P_{\textrm{a}}\text {O}_2$ plateau, but at the cost of shifting the collapse point to higher values of the metabolic demand. The problem remains of finding parameters that can shift the collapse point without elevating the eupneic plateau.

Another parameter that could possibly be affected by COVID-19 infection is hematocrit (hemoglobin concentration). Increased hematocrit (polycythemia) is one phenotypic response observed in individuals who relocate from sea level to extreme high altitude environments for a prolonged period of time (Winslow and Cassinelli 1987; Beall et al. 1990).

### Increasing hemoglobin concentration shifts the $P_{\textrm{a}}\text {O}_2$ collapse point to lower *M* values while maintaining eupneic plateau height

Finally, we considered variation of the parameter [Hb] representing the hematocrit, i.e., the concentration of hemoglobin within the blood, which was set to 150 g/l in the original 7D-O2 model. Figure 5b shows that *increasing* [Hb] within the model *lowers* the collapse threshold of the $P_{\textrm{a}}\text {O}_2$ versus *M* curve, while maintaining a hypoxemic plateau around 80 mmHg. A 33% increase in [Hb] shifts the collapse point to a similar *M* value as the original 7D-O2 model, consistent with the hypothetical silent hypoxemia $P_{\textrm{a}}\text {O}_2$ vs *M* curve shown in Fig. 2d. Further increases in [Hb] yield collapse points with even lower *M* values, consistent with the hypothetical silent hypoxemia $P_{\textrm{a}}\text {O}_2$ vs *M* curve shown in Fig. 2c. See Fig. 9b in Appendix A.2 for the effect of varying [Hb] with all other parameters set to their original 7D-O2 model values.

Thus, we will consider the model with [Hb]=250 (the second curve from the left in Fig. 5b) as a our working model for silent hypoxemia, and analyzed the model dynamics for simulations in the plateau region and in response to increases in metabolic demand.

Figure 6 compares simulations of the silent hypoxemia model (blue traces) and the original 7D-O2 normoxia model (red traces) for different values of the metabolic demand parameter *M*, generated as follows. First, the corresponding model is simulated with $M=0.8\times 10^{-5}\text {ms}^{-1}$ for two minutes of simulated time, to establish baseline initial conditions on the eupneic limit cycle. Then, the value of *M* is changed to the value shown on the horizontal axis (central column: panels b, e, h, k) and the simulation is run for another 10 min of simulated time. For the conditions shown in detail in the left column ($M=0.4\times 10^{-5}\text {ms}^{-1}$, panels a, d, g, j), this duration is sufficient to effectively remove transient behavior. For the conditions shown in the right column ($M=0.97\times 10^{-5}\text {ms}^{-1}$, panels c, f, i, l), the transient effects are still visible for the silent hypoxemia curves.^3^

Figure 6a shows voltage traces in the plateau region ($M=0.4\times 10^{-5}$ ms^-1^) for both the silent hypoxemia model (blue) and the original 7D-O2 normoxia model (red). The frequency of bursting is similar in the two models (Fig. 6b), but there are a few more spikes per burst in the hypoxemia model (Fig. 6d,e). This leads to slightly more vigorous lung expansions in the hypoxemia model (Fig. 6g); however, the levels of oxygen in the blood remain substantially lower (Fig. 6j). As the metabolic demand is increased, the frequency of bursting in the hypoxemia model becomes much faster than in the normoxia model (Fig. 6b), and there are substantially fewer spikes per burst (Fig. 6e). This type of bursting activity leads to more frequent but less vigorous lung expansions and ultimately respiratory collapse at lower levels of metabolic demand in the hypoxemia model compared to the normoxia model (Fig. 6e,k). For comparison, panels (c, f) show voltage traces for a metabolic demand value $M=0.97\times 10^{-5}\text {ms}^{-1}$ for which the SH model’s breathing pattern has moved past the point of collapse, while the original 7D-O2 model maintains steady eupneic breathing; note the dramatically reduced mean $P_{\textrm{a}}\text {O}_2$ values in panel (l).

Figure 6h plots minute ventilation (MV) as a function of metabolic demand *M* for the original 7D-O2 normoxia model (red) and the silent hypoxemia model (blue). MV is a well-established clinical measure of respiratory performance, and is defined as the net volume of respired air. MV is approximately six liters per minute in normal, resting adults, and typically increases with modestly increasing metabolic demand. For excessively high demand, MV shows nonmonotonic behavior in our model, first increasing and then rapidly decreasing. For our model systems, we define MV as the net inspired air per breath (maximum lung volume minus minimum lung volume), divided by the breath cycle duration. For the original model parameters, MV begins near 3 l/min at low metabolic effort ($M=0.2\times 10^{-5}\text {ms}^{-1}$), and increases gradually to approximately 12 l/min at intermediate effort ($M\lesssim 1.2\times 10^{-5}\text {ms}^{-1}$) before collapsing to near zero at excessively high effort ($M \gtrsim 1.2\times 10^{-5}\text {ms}^{-1}$). In contrast, for the silent hypoxemia model, MV begins with slightly elevated values, relative to the normoxia model, climbs gradually while remaining slightly above the normoxia curve, until suddenly collapsing at $\text {MV}\approx 1.0\times 10^{-5}\text {ms}^{-1}$. For comparison, panels (I,L) shows lung volume and $P_{\textrm{a}}\text {O}_2$, respectively, for the higher demand value $M=0.97\times 10^{-5}\text {ms}^{-1}$. The traces show the ongoing transient decline of the respiratory pattern in the SH model, toward full tachypneic collapse, after ten minutes of elevated metabolic demand.

Having established our working model for silent hypoxemia, we next exploit the relative simplicity of the model to investigate the underlying mechanism by which changing hematocrit shifts the collapse point along the *M*-axis.

### Dimension reduction via fast–slow analysis shows varying hematocrit levels has similar effects in the model

Fast–slow dissection is a principled approach to understanding the behavior of dynamical systems involving variables with disparate timescales (Fenichel 1979; Rubin and Terman 2002). Let the vector $\textbf{x}$ represent the fast variables and let the scalar *y* be the slow variable in the two-timescale system

1 $$\begin{aligned} \frac{\textrm{d}\textbf{x}}{\textrm{d}t}&=\textbf{f}(\textbf{x},y) \end{aligned}$$

2 $$\begin{aligned} \frac{\textrm{d}y}{\textrm{d}t}&=\epsilon g(\textbf{x},y) \end{aligned}$$

where $\epsilon \ll 0$ is a small parameter. Suppose (1), the fast subsystem, has either a stable fixed point $\textbf{x}=\textbf{x}_{\textrm{fp}}(y)$ or else a stable limit cycle solution with period *T*(*y*), which we write as $\textbf{x}=\gamma (y,t)=\gamma (y,t+T(y))$, for each value of *y* in the relevant range. The value of the fixed point, or the shape and period of the limit cycle trajectory, may depend on *y*. In the limit of small $\epsilon$, the variable *y* given by (2), the slow subsystem, is approximately constant. Then (2) may be written in terms of rescaled (slow) time $\tau =\epsilon t$ as

3 $$\begin{aligned} \frac{\textrm{d}y}{\textrm{d}\tau }=\bar{g}(y) \end{aligned}$$

where $\bar{g}(y)$ represents the average effect of the fast subsystem on the slow subsystem, at a given value of the slow variable *y*:

4 $$\begin{aligned} \bar{g}(y)={\left\{ \begin{array}{ll} g(\textbf{x}_{\textrm{fp}}(y),y),&{}\text {fixed point case;}\\ \frac{1}{T(y)}\int _0^{T(y)}g(\gamma (y,t),y)\,\textrm{d}t,&{}\text {limit cycle case.} \end{array}\right. } \end{aligned}$$

When the timescales of *y* and $\textbf{x}$ are sufficiently separated, the dynamics given by (3) provide a lower dimensional approximation of the full system (1)–(2). We previously determined $P_{\textrm{a}}\text {O}_2$ to be the slowest dynamical variable in the 7D-O2 model (Diekman et al. 2017). Identifying the slow variable *y* with $P_{\textrm{a}}\text {O}_2,$ we applied fast–slow dissection as described above.

**Fig. 7:**
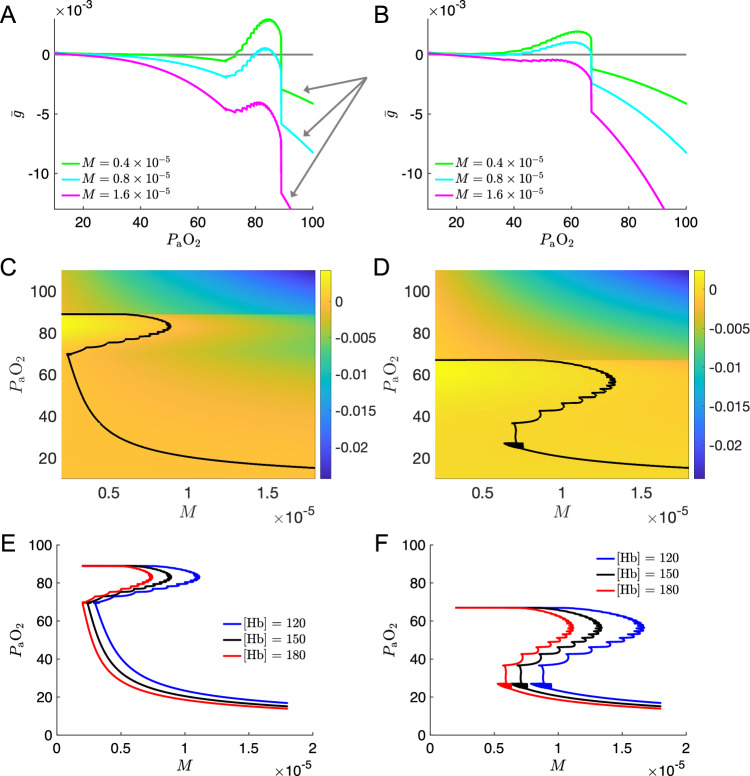
Varying hemoglobin concentration shifts the collapse point in a reduced model obtained from fast–slow decomposition. **a,b** Rate of change of $P_{\textrm{a}}\text {O}_2$ ($\bar{g}$) of averaged slow subsystem (Eq. (3)) for $M=.4\times 10^{-5}\text {ms}^{-1}$ (green), $M=.8\times 10^{-5}\text {ms}^{-1}$ (blue) and $M=1.6\times 10^{-5}\text {ms}^{-1}$ (magenta) for the models with normoxia (panel **a**) and silent hypoxemia (panel **b**) chemosensory parameters. Gray arrows in **a** indicate where $P_{\textrm{a}}\text {O}_2$ lies in a range for which the fast subsystem sits in the quiescent state. **c,d** Heat map showing $\bar{g}$ as a function of $P_{\textrm{a}}\text {O}_2$ and *M* for the models with normoxia (panel **c**) and silent hypoxemia (panel **d**) chemosensory parameters. Black curves: fixed points ($\bar{g}=0$) of the averaged slow subsystem (3). **e,f** Fixed point ($\bar{g}=0$) curves for three levels of [Hb] (mmHg) for the normoxia (panel **e**) and silent hypoxemia (panel **f**) chemosensory parameters

Figure 7 shows the averaged rate of change of the slow subsystem, $\bar{g}$, defined by Eq. (4) for the original 7D-O2 model and the SH model. Panels **a** (redrawn from Diekman et al. (2017)) and **b** show the phase line corresponding to the one-dimensional reduced system (3), which specifies the approximate rate of change of $P_{\textrm{a}}\text {O}_2$ as a function of $P_{\textrm{a}}\text {O}_2$.

The curves in panels a, b have a detailed structure related to the bursting, beating and quiescent regimes of the original BRS model (Butera et al. 1999a). When $P_{\textrm{a}}\text {O}_2$ is in an intermediate range, roughly 70–90 mm Hg in the approximate reduced model, the fast subsystem (1) is in the bursting regime, which efficiently drives gas exchange in the lungs, so that more O2 enters the blood stream than leaves it ($\bar{g}>0$) provided *M* is not too large (green and blue curves, Fig. 7a). Within the eupneic range, the $\bar{g}$ versus $P_{\textrm{a}}\text {O}_2$ curve has a scalloped shape due to the addition of spikes to the bursting pattern as $P_{\textrm{a}}\text {O}_2$ increases. When $P_{\textrm{a}}\text {O}_2$ is below the eupneic range, $g_{\textrm{tonic}}$ increases, forcing the fast subsystem into the steady spiking or “beating” regime, which leads to inefficient gas exchange in the lungs and low minute ventilation. Under these conditions, less O2 enters the blood stream than leaves it ($\bar{g}<0$). When $P_{\textrm{a}}\text {O}_2$ is above the eupneic range, i.e., $P_{\textrm{a}}\text {O}_2\gtrsim 90\,$ mm Hg, $g_{\textrm{tonic}}$ decreases sufficiently that the fast subsystem enters the quiescent state, as described in Butera et al. (1999a). That is, the voltage and gating variables enter a stable fixed point corresponding to a steady resting potential. Under these circumstances, no new oxygen enters the blood stream; meanwhile, O2 leaves in proportion to $P_{\textrm{a}}\text {O}_2$, so the level curves of $\bar{g}$ decrease rapidly. Referring to Eqs. (20)–(25), we see that if the slow variable $y=P_{\textrm{a}}\text {O}_2$ is held constant in a range where the fast subsystem enters the quiescent state then the expression for $\bar{g}$ simplifies to

5 $$\begin{aligned} \bar{g}(y)&=-\frac{M\zeta \left( \beta _{\textrm{O}_2} \, y + \eta \, \text {SaO}_2(y) \right) }{\zeta \left( \beta _{\textrm{O}_2} +\eta \frac{\partial {\text {SaO}_2}}{\partial P_{\textrm{a}}\text {O}_2}(y)\right) } \end{aligned}$$

6 $$\begin{aligned}&\approx -M\frac{\text {SaO}_2(y)}{\text {SaO}_2'(y)}+\mathcal {O} \left( \beta _{\textrm{O}_2}\right) \text {, as }\beta _{\textrm{O}_2}\rightarrow 0, \end{aligned}$$

giving the smooth descending curves above $P_{\textrm{a}}\text {O}_2\gtrsim 90$ mm Hg (see Fig. 7a, arrows). In the second line, we have used the fact that $\beta _{\textrm{O}_2}\ll 1$. We discuss this small parameter further below. In the SH model (Panel b), a similar structure is apparent, but is shifted to the left along the $P_{\textrm{a}}\text {O}_2$ axis.

The curves showing when $P_{\textrm{a}}\text {O}_2$ will increase ($\bar{g}>0$) and decrease ($\bar{g}<0$) provide a simplified explanation of the collapse from eupnea to tachypnea through a saddle-node bifurcation in the slow subsystem. In Fig. 7a, b, zero-crossings of $\bar{g}$ with positive and negative slopes correspond to unstable and stable fixed points of the slow subsystem, respectively. For the 7D-O2 model (Panel a), we can observe the following (cf. Diekman et al. (2017)). When $M=0.4\times 10^{-5}$ ms^-1^ (green curve), the system has a stable fixed point corresponding to eupneic bursting ($P_{\textrm{a}}\text {O}_2$=89 mmHg), a stable fixed point corresponding to tachypneic spiking ($P_{\textrm{a}}\text {O}_2$=41 mmHg), and an unstable fixed point ($P_{\textrm{a}}\text {O}_2$=74 mmHg). When $M=0.8\times 10^{-5}$ ms^-1^ (blue curve), the system still has two stable fixed points, but the stable eupneic point ($P_{\textrm{a}}\text {O}_2$=87 mmHg) and the unstable fixed point ($P_{\textrm{a}}\text {O}_2$=80 mmHg) have moved closer together. Further increases in *M* lead to a saddle-node bifurcation in which the stable eupneic point and the unstable fixed point collide and disappear, leaving only the tachypneic fixed point. For example, when $M=1.6\times 10^{-5}$ ms^-1^ (magenta curve), the system has only 1 fixed point, which corresponds to stable tachypneic spiking ($P_{\textrm{a}}\text {O}_2$=17 mmHg). For the SH model (panel b), as in panel a the system again has three fixed points for $M=0.4\times 10^{-5}$ ms^-1^ and only 1 fixed point for $M=1.6\times 10^{-5}$ ms^-1^; the qualitative behavior is the same, although the value of *M* at which the saddle-node bifurcation occurs is different.

Panels c, d of Fig. 7 show $\bar{g}$ as a function of both blood oxygen ($P_{\textrm{a}}\text {O}_2$) and metabolic demand (*M*), for the reduced system (4) in the 7D-O2 model (panel c) and the SH model (panel d). The black curve in each panel shows the location ($P_{\textrm{a}}\text {O}_2$ value) of fixed points ($\bar{g}=0$) in the averaged slow subsystem as a function of metabolic demand *M*. The heatmap colors indicate the value of $\bar{g}$. For the 7D-O2 model (panel c), at $M=0.25\times 10^{-5}$ ms^-1^, the lower stable branch and unstable middle branch collide and these fixed points are destroyed in a saddle-node bifurcation (SN_1_), leaving only the stable upper branch for $M<\textrm{SN}_1$. Similarly, at $M=0.88\times 10^{-5}$ ms^-1^, the upper stable branch and unstable middle branch collide in another saddle-node bifurcation (SN_2_), leaving only the stable lower branch (tachypnea) for $M>\textrm{SN}_2$. Panel d shows qualitatively similar behavior for the SH model, but the “eupneic” region is shifted to lower $P_{\textrm{a}}\text {O}_2$ values, and the $\bar{g}=0$ curve is shifted to higher values of *M* for corresponding values of [Hb]. Panels e, f illustrate how the curve of fixed points shifts to the right as [Hb] is decreased (blue) and to the left as [Hb] is increased (red) for the normoxia (panel e) and silent hypoxemia (panel f) chemosensory parameters.

### Why does changing [Hb] shift $\bar{g}$ along the $P_{\textrm{a}}\text {O}_2$ axis?

Reexamining the detailed model equations (§A) we see that the metabolic demand parameter *M* occurs only in equation (23). We further note that equation (23) contains a small parameter, namely the Henry’s Law constant ($\beta _{O_2}$) representing the solubility of oxygen in the blood, in the absence of hemoglobin. For the physiologically realistic values chosen in the original model, $3\approx \beta _{\textrm{O}_2} \, P_{\textrm{a}}\text {O}_2\ll \eta \, \text {SaO}_2\approx 150$, in appropriate units. Given the form of (23), it is clear that setting $\beta _{\textrm{O}_2}\approx 0$ is a small (regular) perturbation of the dynamics. Neglecting this small parameter, we see that the flux of oxygen from the blood to the tissue is mainly driven by the product $M\times \eta$ of the metabolic demand parameter *M* and the hematocrit (concentration of hemoglobin) parameter $\eta$. Therefore, the model dynamics are (approximately) invariant to any rescaling $M\rightarrow \gamma M$, $\eta \rightarrow \eta /\gamma$, i.e., any rescaling of *M* and $\eta$ that leaves the product $M\eta$ constant. We thus may predict that running the model with $\eta$ increased and *M* reduced in proportion would shift a $P_{\textrm{a}}\text {O}_2$-versus-$\ln (M)$ curve to the left with little deformation. Thus, if (in the model) the results of COVID-19 infection lead to an increase in a patient’s hematocrit level, for instance, through hypoxia-driven polycythemia, together with the parameter changes necessary to lower the eupneic $P_{\textrm{a}}\text {O}_2$ plateau as in Fig. 5b, we might expect to recover a net shift of the $P_{\textrm{a}}\text {O}_2$-v-*M* curve down and to the left, as suggested in Fig. 1 panel e.

**Fig. 8:**
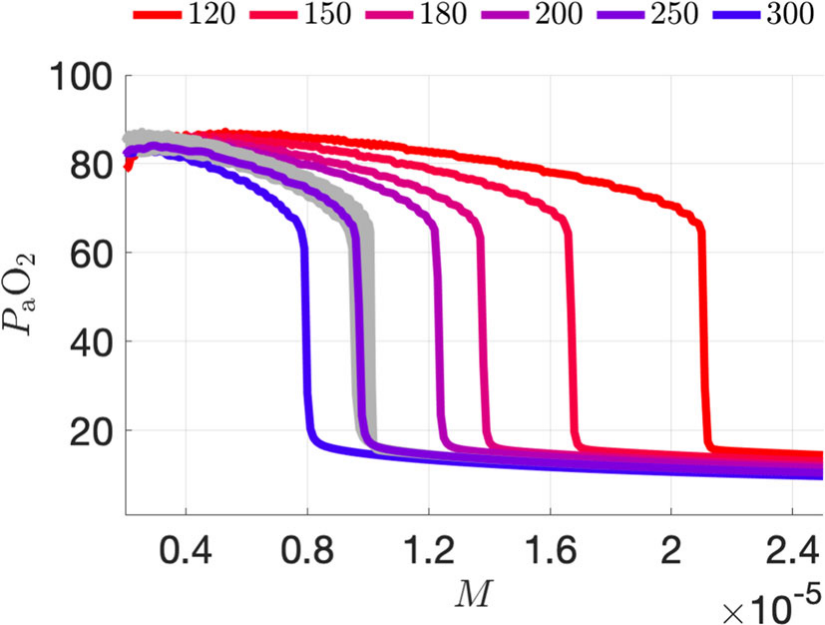
Gray average $P_{\textrm{a}}\text {O}_2$ versus M curves depict the mapping of the curves for 5 different values of hemoglobin concentration ($\mathrm{[Hb]}=120$, 180, 200, 250, and 300) to $\mathrm{[Hb] = 150}$ by multiplying each curve by the ratio [Hb]/150

**Fig. 9:**
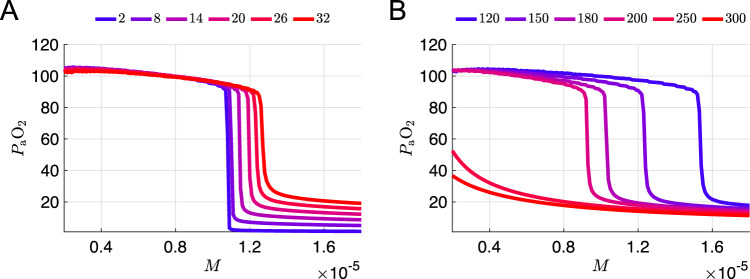
Sensitivity of $P_{\textrm{a}}\text {O}_2$ versus *M* curves to variation of hemoglobin parameters. In both panels, all parameters are set to their values from the original 7D-O2 model except for the parameter being varied. **a** Average $P_{\textrm{a}}\text {O}_2$ vs *M* curves for various hemoglobin binding affinities *K* (including the original value $K=26$ mmHg). Color scale maps the lowest and highest maximum $P_{\textrm{a}}\text {O}_2$ values to blue and red, respectively. **b** Average $P_{\textrm{a}}\text {O}_2$ versus *M* curves for 6 different values of hemoglobin concentration [Hb] (including the original value [Hb] = 250 gm L^-1^) with the same color scale as in (**a**)

It is natural to conjecture that a similar rescaling might be observed in the full system, since the small-parameter argument above applies equally well to the full seven-dimensional model. Figure 8 confirms this conjecture via simulations of the full model using several [Hb] levels. This figure replots the curves from Fig. 5b (colors from blue to red) together with rescaled versions of the figures (gray traces) obtained by multiplying each curve’s abscissa by the ratio [Hb]/150, while keeping its original ordinate. As soon in the figure, the rescaled curves, plotted in gray, collapse onto approximately a common curve.

“Appendix A.2” shows that changing the hematocrit level in the original 7D-O2 model has qualitatively similar effects to those illustrated here.

## Discussion

In this study, we applied a previously published model to the problem of silent hypoxemia seen in a subset of COVID-19 patients. While our model is highly simplified, it can nevertheless be useful for testing hypotheses (about the model) and for generating novel hypothesis relevant to the clinic—although (we hasten to note) we do not make recommendations for clinical *practice*. We consider a simplified breathing model even though more complicated models are available (Molkov et al. 2017). In general, the complexity of a biophysical model should be related to the research question the model is used to address (Thorburn 1918; Koch and Segev 1998; Carnevale and Hines 2006; Levenstein et al. 2023). Simple models often provide useful tools for understanding the behavior of control systems. In the literature, one may find reduced models of the central controller for breathing that are less representative of preBötzinger complex neurons than our version of the Butera–Rinzel–Smith model (Khoo et al. 1982; Khoo 2000; Cheng et al. 2010). Thus, our intention here has been to develop a model that is as reduced as possible, yet still able to recapitulate changes in control of breathing that can be seen in a given disease condition. Our goal has *not* been to capture the full complexity of the rostroventrolateral medulla and the entirety of the brainstem breathing control network. However, we have included aspects of the control circuitry that are important in any whole-body model of breathing control. These include the closed-loop feedback, which represents chemosensation (of oxygen levels via $P_{a}O_2$) and mechanosensory inputs, in an abstracted form at least, via the lung volume component of the model. Because we see responses to changes in breathing patterns that recapitulate many of the features observed in COVID-19 patients in a clinical setting, we see our model as a useful tool for exploring the overall control system for breathing. Our ultimate goal will be to increase the complexity of our model to more closely resemble the architecture and diversity of the brainstem control circuitry. However, that is beyond the scope of this manuscript.

In order to generate hypotheses about silent hypoxemia, we chose to work with a conductance-based CPG model with O_2_ chemosensation as the sensory feedback pathway closing the control loop. To our knowledge, our previously published model (Diekman et al. 2017) is the only model meeting these criteria and our goal here was to extend that model to address a relevant clinical problem. Aspects of our model have been experimentally validated (Diekman et al 2022; Diekman et al. 2018). However, as with any computational model, our model is not designed to encompass all aspects of the respiratory control system. Despite its limitations, the model suffices to generate hypotheses that can be tested in animal models and by the clinical community.

For the original model as presented in Diekman et al. (2017), the $P_{\textrm{a}}\text {O}_2$-vs-*M* curve shows a plateau near 100 mm Hg (normoxia) that collapses to a critically hypoxic state when *M* increases past a high threshold (Fig. 2a). The work we report here focuses on expanding the original parameters to investigate possible mechanisms of SH. Changing these parameters allowed us to monitor the height of the normoxia plateau, and the location of the collapse point. We hypothesized that altered chemosensory input to the carotid bodies and, eventually, to the NTS and the rest of the breathing control circuitry, is a key factor in silent hypoxemia. However, our simulation results suggest that while changes in chemosensivity may play a role in silent hypoxemia, changes in metabolism and oxygen carrying capacity may have greater relevance for replicating the respiratory collapse seen in these patients. Specifically, altered chemosensitivity can create a hypoxemic plateau region ($\text {SaO}_2<90$ mmHg) for a broad range of metabolic demand levels ($M=0.4$ to 1.5$\times 10^{-5}$ ms^-1^, see blue curves in Fig. 3b). When hemoglobin concentration is then increased, moderate levels of metabolic demand ($M=0.8$ to 1.0$\times 10^{-5}$ ms^-1^) lead to complete respiratory collapse ($\text {SaO}_2<60$ mmHg, see blue and purple curves in Fig. 3f).

Our hypothesis was based on the premise that O_2_ sensing is the key factor in SH. Canonically, it has been suggested that CO_2_ is a primary driver for dyspnea (Cherniack and Altose 1987; Chonan et al. 1990; Guyenet and Bayliss 2022), but there is evidence that both hypoxia and hypercapnia equivalently drive the sensation of air hunger (Moosavi et al. 2003). However, clinical case and cohort studies show that patients with SH are not hypercapnic (Chandra et al. 2020; Alamé et al. 2022). This suggested to us that dysregulation of O_2_ sensation is a key contributor to the issues seen in SH. We tested this hypothesis by changing O_2_ sensitivity in the model at the level of the carotid bodies and NTS as well as evaluating whether those changes could reproduce the SH phenotype.

Complicating factors for these patients include comorbidities that show correlation with poor outcome in patients with COVID-19. These comorbidities include obstructive sleep apnea (OSA), chronic obstructive pulmonary disease (COPD), cardiovascular disease (including hypertension or heart failure) among others. Patients suffering from these diseases often develop polycythemia—an increase in the hemoglobin and hematocrit to adaptively increase the O_2_ carrying capacity of the blood. High-altitude populations are well adapted to chronic hypoxia and typically have a higher hematocrit in Andean populations versus Himalayan high-altitude dwellers (Beall and Reichsman 1984), likely due to different adaptation mechanisms. However, subjects with cardiovascular disease (Valeanu et al. 2022), obstructive apnea (Rha et al. 2022), and familial hyperlipidemia (Paquette et al. 2021) also show increased hematocrit.

One consequence of pumping thicker blood is to increase the metabolic demand, even during rest. As we show in our results, increasing metabolic demand increases the likelihood of respiratory collapse. Somewhat paradoxically, with an increased oxygen carrying capacity, the patient may be less able to compensate for the worsening $P_{\textrm{a}}\text {O}_2$ and a critical tipping point for metabolic demand is reached where respiratory efforts are insufficient to keep up with demand. We have not yet seen any report documenting changes in hematocrit in COVID-19 patients who exhibit silent hypoxemia. Based on our modeling results, we would predict that these patients may show increased hematocrit levels. In support of our prediction, a recently published study (Choi et al. 2022) showed that higher blood viscosity was associated with an increase in mortality in COVID-19 patients. Obtaining this kind of data should be possible for patients admitted to the intensive care unit and should be a priority for future investigation.

Angiotensin-converting enzyme 2 (ACE2) is expressed in the lungs, carotid bodies, and respiratory region of the brainstem, and is likely the vector by which the SARS-CoV-2 virus invades the carotid bodies and/or the NTS, thereby potentially contributing to silent hypoxemia. High ACE2 levels also occur in the most vulnerable target organ systems seen in COVID-19 (elevated expression levels occur in lung, heart, ileum, kidney, and bladder (Zou et al. 2020)). Since ACE2 expression is very high in the lungs, and since diffuse alveolar damage, bronchopneumonia, and alveolar hemorrhage are common in COVID-19 (Mauad et al. 2021), it seems reasonable to hypothesize that the decrease in gas exchange across the alveolar membranes within the lung can alter not just the O_2_ carrying capacity, but also increase metabolic demand for perfusion of the damaged lung. It may be of value to assess differences in mitochondrial activity in lung cells from normal and COVID-19 patients, or in animal models that have used SARS-CoV-2 or spike protein (now commercially available) to mimic the lung damage seen in human patients. Such experiments would provide data concerning cellular metabolism and give us greater understanding of the impact COVID-19 has on metabolic demand at all tissue levels.

Lack of dyspnea (breathing discomfort) in patients arriving at already overcrowded emergency rooms leads to triaging patients who are not in obvious respiratory distress, when in fact these patients often have reduced oxygen saturation (Bertran et al. 2020). Perhaps the greatest mystery that remains unresolved is why dyspnea is not typically seen in patients exhibiting silent hypoxemia. Sensory perception is subjective and can vary with a host of factors that include sex, socioeconomic background, and ethnicity (Green et al. 2003; Reynolds Losin et al. 2020; de Araújo Palmeira et al. 2011). There is some controversy about these correlates but they may be underlying factors that influence the reporting of silent hypoxemia. Once again, some demographic data is available concerning COVID-19 infection, mortality, and morbidity, but this information has not yet been correlated with silent hypoxemia. Ideally, demographic factors should be reported along with other patient data to better understand the incidence and severity of silent hypoxemia and dyspnea.

Patients with COVID-19 are also subject to mitochondrial dysregulation that contributes to severity and lethality. Mitochondrial function is impacted by the “cytokine storm,” a hallmark of the immune response to COVID-19. Thus, upregulation of cytokine release in the context of comorbidities that increase inflammation, including metabolic syndrome, obesity, type 2 diabetes, and increasing age—in addition to the lung and cardiovascular diseases mentioned above, are all associated with mitochondrial dysfunction (Moreno Fernández-Ayala et al. 2020; Grossini et al. 2021; Wang et al. 2022). SARS-CoV-2 infection causes multi-system changes at transcriptomic, proteomic, and metabolomic levels, altering normal cellular metabolism and changing mitochondrial respiration (Wang et al. 2022). Disruption of normal mitochondrial function can result in an increase in reactive oxygen species (ROS) further exacerbating inflammation and increasing the likelihood of poor outcomes (Saleh et al. 2020). The “long COVID” phenomenon may be related to redox imbalance, which may be exacerbated by COVID-induced changes in mitochondria (Paul et al. 2021; Singh et al. 2020) and, ultimately, fatigue related to metabolic impairment. Our results suggest that there is a delicate balance between metabolic demand changes and respiratory failure. One can easily speculate that reduction in available oxygen in concert with an increase in metabolic demand as the virus takes over cellular machinery to produce more viral particles can result in a point of critical failure. However, it is not intuitively obvious that greater O_2_ carrying capacity results in metabolic collapse. Our model treats the relationship between oxygen carrying capacity and metabolic demand simplistically and we have not incorporated blood viscosity changes and their impact on cardiovascular function, particularly cardiac output as the key factor driving tissue perfusion.

Mitochondrial dysregulation due to COVID-19 results in pronounced impacts on blood coaguability (Hai-Han et al. 2020), handling of reactive oxygen species (ROS)—increased by the cytokine storm associated with COVID-19 (Saleh et al. 2020), calcium homeostasis (Yang et al. 2021), iron homeostasis (Vlahakos et al. 2021; Abobaker 2020), as well as cellular metabolism (Henry et al. 2020; Booth et al. 2021). COVID-19 significantly impacts each of these aspects of mitochondrial function and this results in altered ability to respond to cytokine induced ROS changes, as well as reduced metabolic capacity. All of these alterations in mitochondria function contribute to changes in metabolic demand and it may be that, regardless of the O_2_ concentration in the blood, the mitochondria are not able to utilize the available O_2_. In this study, we have shown that changes in metabolic reserve—particularly an impaired ability to meet metabolic demand in the context of respiratory function—can result in collapse of respiration contributing to death. These changes are also most likely to be key factors in patients presenting with SH.

One way to test the impact of COVID-19 on mitochondrial function would be to assay mitochondria obtained from tissue biopsies of COVID-19 patients or through animal models. Testing mitochondrial metabolism would be easier than using stress tests or cycle ergometry to determine metabolic load and ventilation/perfusion changes. Whole body tests would be problematic in COVID-19 patients and put them at greater risk for respiratory collapse. As long COVID has become better described, central nervous system (CNS) involvement and increased chronic inflammation are seen as sequelae that may continue to alter metabolism and mitochondrial function (Stefano et al. 2021). Further research is needed to determine if these effects are exacerbated by persistent metabolic impairment and whether symptoms like cognitive fog depend on CNS mitochondria and ROS handling problems. The relationship between changes in overall metabolic demand and cellular level metabolism have not yet been explored in COVID-19 patients. This is an important area for investigation because, while the metabolic demand required to pump more viscous blood (Choi et al. 2022) may selectively impact the cardiovascular system the most, metabolic demand may be increased systemically based on the diffuse organ involvement seen in these patients.

In addition to the model limitations we mentioned above, we realize that our model represents a very reduced number of the elements in the central pattern generator and pattern formation network for breathing control. The brainstem network includes hundreds of neurons that participate in each breath (Wang et al. 2014; Carroll and Ramirez 2013), and we have simplified this relatively complex circuit for the sake of rapid simulation time to test our hypotheses about SH. This heavily reductionist treatment of the brainstem network is an obvious limitation to simulation of the interacting populations of respiratory neurons and makes it difficult to interrogate the precise mechanisms by which respiratory collapse occurs in SH. Three examples include, (1) we have not included CO_2_ sensing in our model due to the high diffusion rates of CO_2_ when compared to O_2_ in the lung (West 2008) and evidence showing that CO_2_ is $\le$ 35 mmHg in patients presenting with silent hypoxemia (SH) and minimal tachypnea (Chandra et al. 2020; Alamé et al. 2022; 2) we do not explicitly include rapidly adapting (RAR) or slowly adapting (SAR) lung mechanoreceptors in the model—lung volume is present in the model and reproduces inspiratory drive in much the same way that SARs do *in vivo*; (3) the lack of a specific mechanism for understanding the relationship between blood viscosity, number of red cells, oxygen carrying capacity, and changes in metabolic demand.

Previously, we have demonstrated that increasing extracellular [K^+^] resulted in a progressive increase in respiratory rhythm that showed periodic, multi-periodic, quasi-periodic, and finally chaotic rhythmic patterns (Del Negro et al. 2002). As excitability increased, the disruption to eupneic breathing would result in impaired gas exchange *in vivo*. Thus, there is precedent for increasing excitability in the respiratory network resulting in a kind of “depolarization blockade” of normal breathing and a cessation of gas exchange that then results in a precipitous fall in $P_{\textrm{a}}$O_2_. We described experiments related to this concept in our prior work (Diekman et al. 2017). Because we have previously shown these transitions are gradual and occur over a wide range of excitability changes, it makes sense to assume that there may be a more gradual progression of the “respiratory collapse,” but we do not yet have clinical data showing how the collapse evolves to the point of need for ventilatory support.

Our model predicts changes in oxygen handling and metabolism in silent hypoxemia patients. As an obvious next step, we call for data to be collected on hematocrit in animal models of COVID-19 infection and COVID-19 patients, with the inclusion of metabolism and mitochondrial function tests. In addition, we believe the following measures may have untapped predictive value: minute ventilation, oxygen saturation, and breathing frequency. We speculate that some combination of these quantities, if measured on entry to the ER, could help predict the need for ventilator support in the subsequent 48 h. Finally, we emphasize that there is a need for incorporating oxygen handling dynamics into more sophisticated state-of-the-art respiratory control models, most of which currently focus on CO_2_ and hypercapnea (Molkov et al. 2017).

## Appendix A

### Detailed model specification

Here, we provide the equations for the 7D-O2 model introduced in Diekman et al. (2017).

*Central pattern generator (CPG)*: A variety of models have been proposed for the central neural circuits generating breathing rhythms, ranging from group-pacemaker networks to individual pacemaker models, and beyond. Here, we adopt the original Butera–Rinzel–Smith (BRS) model (referred to as “model 1” in Butera et al. (1999a)) proposed as a mechanism for bursting pacemaker neurons in the preBötzinger complex. For simplicity, we represent the CPG with a single BRS unit. Thus, our CPG is described by a membrane potential *V* together with dynamical gating variables *n* (a delayed-rectifier potassium ($I_{\textrm{K}}$) channel activation) and *h* (persistent sodium ($I_{\textrm{NaP}}$) channel inactivation). We set two “instantaneous” gating variables $p_\infty$ ($I_{\textrm{NaP}}$ activation) and $m_\infty$ (fast sodium ($I_{\textrm{Na}}$) activation) to be equal to their voltage-dependent asymptotic values. We set the $I_{\textrm{Na}}$ inactivation gate to be equal to $(1-n)$. The model also includes a leak current ($I_{\textrm{L}}$) and a tonic excitatory ($I_{\textrm{tonic}}$) current. In summary:

7 $$\begin{aligned} C\frac{dV}{dt}&= -I_{\textrm{K}}-I_{\textrm{NaP}} -I_{\textrm{Na}}-I_{\textrm{L}}-I_{\textrm{tonic}} \end{aligned}$$

8 $$\begin{aligned} \frac{dn}{dt}&= \frac{n_\infty (V)-n}{\tau _{\textrm{n}}(V)} \end{aligned}$$

9 $$\begin{aligned} \frac{dh}{dt}&= \frac{h_\infty (V)-h}{\tau _{\textrm{h}}(V)} \end{aligned}$$

10 $$\begin{aligned} I_{\textrm{K}}&=g_{\textrm{K}}n^4(V-E_{\textrm{K}}) \end{aligned}$$

11 $$\begin{aligned} I_{\textrm{NaP}}&=g_{\textrm{NaP}}p_\infty (V)h(V-E_{\textrm{Na}}) \end{aligned}$$

12 $$\begin{aligned} I_{\textrm{Na}}&=g_{\textrm{Na}}m_\infty ^3(V)(1-n)(V-E_{\textrm{Na}}) \end{aligned}$$

13 $$\begin{aligned} I_{\textrm{L}}&=g_{\textrm{L}}(V-E_{\textrm{L}}) \end{aligned}$$

14 $$\begin{aligned} I_{\textrm{tonic}}&=g_{\textrm{tonic}}(V-E_{\textrm{tonic}}) \end{aligned}$$

15 $$\begin{aligned} x_\infty (V)&=\frac{1}{1+\exp [(V-\theta _{\textrm{x}})/\sigma _{\textrm{x}}]} \end{aligned}$$

16 $$\begin{aligned} \tau _{\textrm{x}}&=\frac{\bar{\tau }_{\textrm{x}}}{\cosh [(V-\theta _{\textrm{x}})/2 \sigma _{\textrm{x}}]} \end{aligned}$$

with parameters $C=21$ pF, $g_{\textrm{K}}=11.2$ nS, $g_{\textrm{NaP}}=2.8$ nS, $g_{\textrm{Na}}=28$ nS, $g_{\textrm{L}}=2.8$ nS, $E_{\textrm{K}}=-85$ mV, $E_{\textrm{Na}}=50$ mV, $E_{\textrm{L}}=-65$ mV, $E_{\textrm{tonic}}=0$ mV, $\theta _{\textrm{n}}=-29$ mV, $\sigma _{\textrm{n}}=-4$ mV, $\theta _{\textrm{p}}=-40$ mV, $\sigma _{\textrm{p}}=-6$ mV, $\theta _{\textrm{h}}=-48$ mV, $\sigma _{\textrm{h}}=6$ mV, $\theta _{\textrm{m}}=-34$ mV, $\sigma _{\textrm{m}}=-5$ mV, $\bar{\tau }_{\textrm{n}}=10$ ms, and $\bar{\tau }_{\textrm{h}}=10,000$ ms.

it Motor pool activity The output of the CPG is the BRS cell’s membrane potential (*V*), which drives the respiratory muscles through synaptic activation of a motor unit ($\alpha$):

17 $$\begin{aligned} \frac{d\alpha }{dt}&= r_{\textrm{a}}[T](1-\alpha )-r_{\textrm{d}}\alpha \end{aligned}$$

18 $$\begin{aligned} {[}T]&= \frac{T_{\textrm{max}}}{(1+\exp (-(V-V_{\textrm{T}})/K_{\textrm{p}}))}. \end{aligned}$$

Here, $r_{\textrm{a}}=r_{\textrm{d}}=0.001$ mM^-1^ ms^-1^ sets the rise and decay rate of the synaptic conductance. Also, [*T*] represents the neurotransmitter concentration, with parameters $T_{\textrm{max}}=1$ mM, $V_{\textrm{T}}=2$ mV, and $K_{\textrm{p}}=5$ mV (Bard Ermentrout and Terman 2010).

*Lung volume* The output of the motor unit determines the rise and fall of lung volume ($\text {vol}_{\textrm{L}}$):

19 $$\begin{aligned} \frac{d}{dt}(\text {vol}_{\textrm{L}})&= E_1\alpha - E_2 (\text {vol}_{\textrm{L}}-\text {vol}_0). \end{aligned}$$

Here $\text {vol}_0=2$ L is the volume of the unloaded lung, and parameters $E_1=0.4$ L and $E_2=0.0025$ ms^-1^ were chosen so that the lung expansion would remain in a physiologically reasonable range (West 2008). We note that while the low-frequency input of the envelope of CPG burst activity effectively drives changes in lung volume, high-frequency input (such as tonic spiking) does not drive the lung biomechanics effectively. This low-pass filter behavior of the respiratory musculature is analogous to tetanic muscle contraction that occurs in response to high-frequency stimulation of motor nerves (Kandel et al. 1991).

*Lung oxygen* At standard atmospheric pressure (760 mmHg), external air with 21% oxygen content will register a partial pressure of oxygen of $P_{\textrm{ext}}\text {O}_2=149.7$ mmHg. As the lungs expand $\left( \frac{d}{dt}\left[ \text {vol}_{\textrm{L}}\right] >0 \right)$, they draw in external air. Our model makes the simplifying assumption that this fresh air mixes instantaneously with the air already present in the lungs. Therefore, the partial pressure of oxygen in the lung alveoli ($P_{\textrm{A}}\text {O}_2$) increases at a rate given by the pressure difference between external and internal air, and by the lung volume. In contrast, during exhalation $\left( \frac{d}{dt}\left[ \text {vol}_{\textrm{L}}\right] \le 0\right)$, no external air enters the lungs, so the mixing of air stops. During both contraction and expansion of the lung, oxygen moves between the lungs and the blood. The flux of oxygen from the lungs to the blood occurs at a rate determined by the time constant $\tau _{LB}=500$ ms, and by the difference in partial pressure of $\text {O}_2$ between the lungs ($P_{\textrm{A}}\text {O}_2$) and the arterial blood ($P_{\textrm{a}}\text {O}_2$). The rate of change in $P_{\textrm{a}}\text {O}_2$ is thus given by:

20 $$\begin{aligned}&\frac{d}{dt}(P_{\textrm{A}}\text {O}_2)= \frac{P_{\textrm{ext}}\text {O}_2 -P_{\textrm{A}}\text {O}_2}{\text {vol}_{\textrm{L}}}\left[ \frac{d}{dt} (\text {vol}_{\textrm{L}})\right] _+\nonumber \\&\quad \qquad \qquad - \frac{P_{\textrm{A}}\text {O}_2-P_{\textrm{a}}\text {O}_2}{\tau _{\textrm{LB}}} \end{aligned}$$

where the notation $[x]_+$ indicates $\text{ max }(x,0)$.

*Blood oxygen* To represent the change in $P_{\textrm{a}}\text {O}_2$, we write

21 $$\begin{aligned} \frac{d}{dt}(P_{\textrm{a}}\text {O}_2)=\frac{J_{\textrm{LB}}-J_{\textrm{BT}}}{\zeta \left( \beta _{\textrm{O}_2}+\eta \frac{\partial {\text {SaO}_2}}{\partial P_{\textrm{a}}\text {O}_2}\right) }. \end{aligned}$$

Note the fluxes of oxygen from the blood to the tissues $\left( J_{\textrm{BT}}\right)$ and from the lungs to the blood $\left( J_{\textrm{LB}}\right)$ have units of moles of $\text {O}_2$ per millisecond. The term in the denominator converts from units of moles per millisecond (the rate of change of moles of $\text {O}_2$ in the blood) into units of millimeters of mercury per millisecond (rate of change of partial pressure of $\text {O}_2$ in the blood). To calculate the flux $J_{\textrm{LB}}$, we use the ideal gas law $PV=nRT$, where *n* is the number of moles of $\text {O}_2$, $R=62.364 ~ \text {L} ~ \text {mmHg} ~ \text {K}^{-1} ~ \text {mol}^{-1}$ is the universal gas constant, and $T=310$ K is temperature. The resulting flux depends on the difference in oxygen partial pressure between the lungs and the blood:

22 $$\begin{aligned} J_{\textrm{LB}}= \left( \frac{P_{\textrm{A}}\text {O}_2-P_{\textrm{a}}\text {O}_2}{\tau _{\textrm{LB}}}\right) \left( \frac{\text {vol}_{\textrm{L}}}{RT}\right) . \end{aligned}$$

Note that the term $J_{\textrm{BT}}$ accounts for both dissolved and hemoglobin-bound oxygen in the blood:

23 $$\begin{aligned} J_{\textrm{BT}}=M\zeta \left( \beta _{\textrm{O}_2} \, P_{\textrm{a}}\text {O}_2+\eta \, \text {SaO}_2\right) . \end{aligned}$$

The dependence of the hemoglobin saturation ($\text {SaO}_2$) on $P_{\textrm{a}}\text {O}_2$ is given below, see Eqn. (24). Following Henry’s law, we take the concentration of dissolved oxygen in the blood to be directly proportional to $P_{\textrm{a}}\text {O}_2$. The blood solubility coefficient, $\beta _{\textrm{O}_2}=0.03 ~ \text {ml O}_2 ~ \times ~ \text {L blood}^{-1} ~ \text {mmHg}^{-1}$ for blood at 37 degrees C, is the constant of proportionality. The amount of dissolved $\text {O}_2$ at physiological partial pressures ($P_{\textrm{a}}\text {O}_2\approx 80-110$ mmHg) is insufficient to satisfy the body’s metabolic demand for oxygen. Therefore, most of the blood’s stored oxygen is bound to hemoglobin (Hb). Cooperative binding of oxygen to the four binding sites in each hemoglobin molecule leads to a sigmoidal hemoglobin saturation curve:

24 $$\begin{aligned} \text {SaO}_2&=\frac{P_{\textrm{a}}\text {O}_2^c}{P_{\textrm{a}}\text {O}_2^c+K^c} \end{aligned}$$

25 $$\begin{aligned} \frac{\partial {\text {SaO}_2}}{\partial P_{\textrm{a}}\text {O}_2}&= cP_{\textrm{a}}\text {O}_2^{c-1} \left( \frac{1}{P_{\textrm{a}}\text {O}_2^c+K^c}-\frac{P_{\textrm{a}}\text {O}_2^c}{(P_{\textrm{a}}\text {O}_2^c+K^c)^2}\right) . \end{aligned}$$

Here, we take the phenomenological parameters $K=26$ mmHg and $c=2.5$ from Keener and Sneyd (2009).

Our model includes a parameter *M* in Eq. (23) to capture the rate of metabolic demand for oxygen from the tissues, in units of $\textrm{ms}^{-1}$. Equations (21) and (23) include conversion factors $\zeta$ and $\eta$ that depend on the concentration of hemoglobin, $[\text {Hb}]=150 ~ \text {gm} ~ \text {L}^{-1}$, as well as the volume of blood, $\text {vol}_{\textrm{B}}=5 ~ \text {L}$, respectively. The model assumes a molar oxygen volume of 22.4 L. We assume that each fully saturated hemoglobin molecule carries 1.36 ml of $\text {O}_2$ per gram:

26 $$\begin{aligned} \zeta&= \text {vol}_{\textrm{B}}\times \left( \frac{\text{ mole }\ \text{ O}_2}{22,400\ \text{ mL }\ \text{ O}_2}\right) \end{aligned}$$

27 $$\begin{aligned} \eta&=[\text {Hb}] \times \left( \frac{1.36\ \text{ mL }\ \text{ O}_2}{\text{ gm }\ \text{ Hb }}\right) . \end{aligned}$$

**Chemosensation:** Chemosensory feedback from peripheral chemoreceptors in the carotid bodies, carried to brainstem respiratory circuits via the carotid sinus nerve, close the control loop in our model. These receptors detect reductions in $P_{\textrm{a}}\text {O}_2$ and drive the central rhythm generator, as described in more detail in Diekman et al. (2017). We model the nonlinear relationship between carotid chemosensory nerve fiber activity and $P_{\textrm{a}}\text {O}_2$ as a sigmoid saturating function, with the firing rate low until $P_{\textrm{a}}\text {O}_2$ is reduced below a threshold (normally about 100 mm Hg) and then steep firing rate increases as $P_{\textrm{a}}\text {O}_2$ is reduced further (Hlastala and Berger 2001; West 2008). We capture this behavior in our model as a sigmoidal function connecting $P_{\textrm{a}}\text {O}_2$ with the conductance representing external drive to the CPG ($g_{\textrm{tonic}}$):

28 $$\begin{aligned} g_{\textrm{tonic}}=\phi \left( 1-\text{ tanh }\left( \frac{P_{\textrm{a}}\text {O}_2-\theta _g}{\sigma _g}\right) \right) . \end{aligned}$$

Here, $\phi =0.3$ nS, $\theta _g=85$ mmHg, and $\sigma _g=30$ mmHg. This conductance closes the control loop in our respiratory control model, since $I_{\textrm{tonic}}=g_{\textrm{tonic}}(V-E_{\textrm{tonic}})$ is a term in the CPG voltage equation (7).

We numerically integrated the preceding equations using a variable-order, variable-step stiff solver (ode15s in MATLAB).

### Hemoglobin effects given the original model parameters

In this section, we illustrate the effects of changing the parameters controlling the hemoglobin binding curve and the total hemoglobin (hematocrit) in the *original* model, as opposed to the model adjusted by successive parameter shifts (see Sect. 3). Panel a shows the effect of increasing *K*, the hemoglobin binding affinity. Panel b shows the effect of varying [Hb], the hemoglobin concentration. The effects are qualitatively similar to the effects of analogous parameter changes in the silent hypoxemia model.

## Footnotes

1 The oxyhemoglobin dissociation curve gives the steady-state fraction of hemoglobin capacity occupied by oxygen, as a function of oxygen tension in the blood.

2 Model code is available in *ModelDB* at: https://modeldb.science/2015954.

3 The same procedure was used to generate Figs. 3b, 4b, and 5.
